# Transcriptome profiling of immune response to *Yersinia ruckeri* in spleen of rainbow trout (*Oncorhynchus mykiss*)

**DOI:** 10.1186/s12864-021-07611-4

**Published:** 2021-04-22

**Authors:** Di Wang, Simeng Sun, Shaowu Li, Tongyan Lu, Dongfang Shi

**Affiliations:** 1 College of Veterinary Medicine, Northeast Agricultural University, 150030 Harbin, China; 2 Heilongjiang River Fisheries Research Institute, Chinese Academy of Fishery Sciences, 150070 Harbin, China; 3 Key Laboratory of Aquatic Animal Diseases and Immune Technology of Heilongjiang Province, 150070 Harbin, China

**Keywords:** Rainbow trout, *Yersinia ruckeri*, Spleen, Transcriptome, Immune response

## Abstract

**Background:**

*Yersinia ruckeri* is a pathogen that can cause enteric redmouth disease in salmonid species, damaging global production of economically important fish including rainbow trout (*Oncorhynchus mykiss*). Herein, we conducted the transcriptomic profiling of spleen samples from rainbow trout at 24 h post-*Y. ruckeri* infection via RNA-seq in an effort to more fully understand their immunological responses.

**Results:**

We identified 2498 differentially expressed genes (DEGs), of which 2083 and 415 were up- and down-regulated, respectively. We then conducted a more in-depth assessment of 78 DEGs associated with the immune system including *CCR9*, *CXCL11*, *IL-1β*, *CARD9*, *IFN*, *TNF*, *CASP8*, *NF-κB*, *NOD1*, *TLR8α2*, *HSP90*, and *MAPK11*, revealing these genes to be associated with 20 different immunological KEGG pathways including the Cytokine-cytokine receptor interaction, Toll-like receptor signaling, RIG-I-like receptor signaling, NOD-like receptor signaling, and MAPK signaling pathways. Additionally, the differential expression of 8 of these DEGs was validated by a qRT-PCR approach and their immunological importance was then discussed.

**Conclusions:**

Our findings provide preliminary insight on molecular mechanism underlying the immune responses of rainbow trout following *Y. ruckeri* infection and the base for future studies of host-pathogen interactions in rainbow trout.

**Supplementary Information:**

The online version contains supplementary material available at 10.1186/s12864-021-07611-4.

## Background

*Yersinia ruckeri* is a pathogen that can cause enteric redmouth disease (ERM) or yersiniosis, resulting in significant mortality and economic losses associated with the global production of rainbow trout (*Oncorhynchus mykiss*). Rainbow trout are highly susceptible to ERM, although other species of fish can also be affected by this disease [1, 2]. Multiple studies have sought to clarify the immunological responses of fish species to *Y. ruckeri* infection [3, 4]. In one study, Raida et al. determined that very susceptible trout species exhibited a robust and rapid-onset septicemic response to infection associated with the production of high levels of pro-inflammatory cytokines [5]. Similarly, these pro-inflammatory cytokines were also upregulated in the spleen of the vaccinated rainbow trout following *Y. ruckeri* challenge, albeit to a lesser extent than in naïve fish [6]. The spleen is a key secondary lymphoid organ that is thus closely associated with rainbow trout responses to *Y. ruckeri* infection, and significant changes in the expression of splenic immune-related genes have been detected following *Y. ruckeri* challenge [7, 8]. However, no systematic analyses of patterns of rainbow trout splenic gene expression after *Y. ruckeri* infection have been conducted to date.

RNA sequencing (RNA-seq) is a high-throughput approach to analyzing transcriptomes that has frequently been employed in studies of fish species [9]. Several recent studies based on RNA-Seq analysis have explored rainbow trout responses to a range of pathogen types, such as splenic responses to *Aeromonas salmonicida* [10, 11], infectious hematopoietic necrosis virus (IHNV) [12], and *Ichthyophthirius multifiliis* [13]. Such transcriptomic analyses have offered new insights into the etiology of these diseases, and similar studies of *Y. ruckeri* infections may highlight viable approaches for treating or preventing yersiniosis in rainbow trout farming.

As such, we herein conducted a transcriptomic study assessing rainbow trout splenic immune responses to *Y. ruckeri* infection. After identifying infection-related differentially expressed genes (DEGs), we validated a subset of these genes via qRT-PCR and conducted the functional annotation of immune-associated DEGs. Together, our data offer a preliminary insight for future research regarding the immunological mechanisms involved in rainbow trout defensive response against *Y. ruckeri*.

## Results

### RNA-sequencing and data processing

Genes associated with rainbow trout immune response to *Y. ruckeri* infection were identified by assessing spleen samples from YR-infected and control uninfected fish via RNA-sEq. In total, six cDNA libraries were prepared (from 3 per group), and raw data were generated (Table S1) and deposited in the NCBI Sequence Read Archive (SRA) under accession number SRR13014589 ~ SRR13014594.

Following the completion of filtering, 44.07 G bp of clean data were extracted, with over 93.15–93.55 % of the bases reads having a phred quality value ≥ 30 in the non-infected group compared to 92.87–93.43 % in the YR-infected group. These quality scores were consistent with excellent quality data. Reads from these two groups exhibited GC contents of 49.14–49.64 % and 49.00-49.18 %, respectively (Table 1).

**Table 1:** Characteristics of RNA-seq data

Samples	Clean reads (M)	Clean bases (Gb)	GC Content (%)	Q30 (%)
non-infected rainbow trout 1	26.9191	8.0181	49.14	93.18
non-infected rainbow trout 2	23.4555	6.9991	49.64	93.55
non-infected rainbow trout 3	25.5238	7.6123	49.33	93.15
YR-infected rainbow trout 1	22.5961	6.7523	49.18	92.87
YR-infected rainbow trout 2	23.5341	7.0248	49.00	93.43
YR-infected rainbow trout 3	25.7487	7.6694	49.18	93.32
Non-infected group	75.8984	22.6295	49.32	93.29
YR-infected group	72.0286	21.4465	49.12	93.21
Total	147.927	44.076	49.25	93.25

**Fig. 1:**
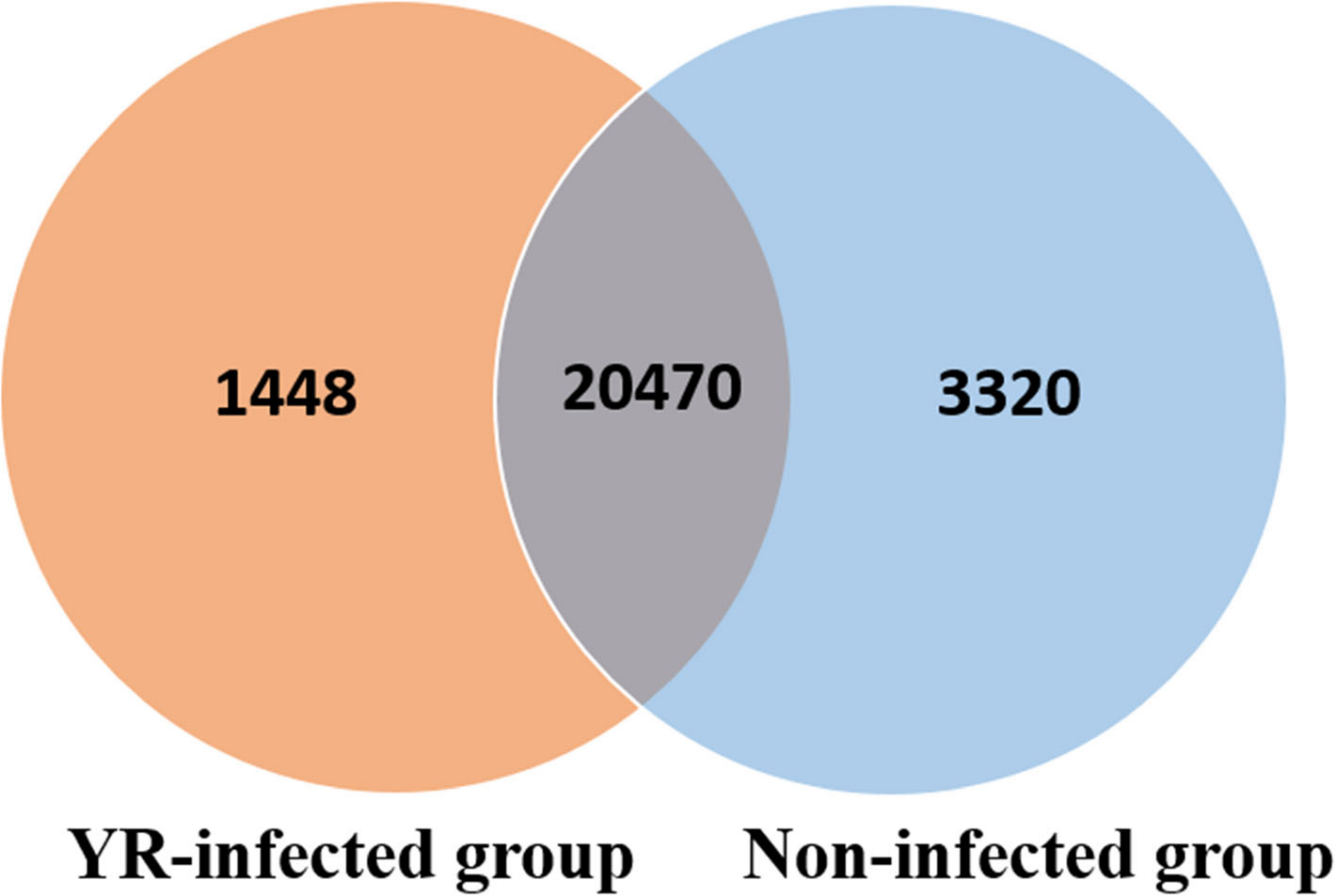
A Venn diagram indicating the numbers of genes detected in YR-infected and uninfected rainbow trout spleen samples

The total number of expressed genes detected in samples from uninfected rainbow trout was slightly higher than that detected in YR-infected rainbow trout (Fig. 1).

### Read mapping to the reference genome

Cleaned reads were mapped to the *O. mykiss* reference genome, with 84.81–85.99 % of these reads ultimately matching perfectly. Over 70 % of reads aligned to exonic regions in each library, of which 78.05–78.24 % in the uninfected group and 78.53–79.11 % in the YR-infected groups mapping to unique reads whereas 6.76–7.38 % in the uninfected group and 6.81–7.17 % in the YR-infected groups mapping to multiple reads. A total of 123.7985 (41.90 %) and 125.0329 (42.32 %) M reads in the uninfected and YR-infected groups mapped to reference genome sense and antisense strands, respectively (Table 2). Besides, some new genes were detected and classified with the NR, Swiss-Prot, GO, COG, KOG, Pfam, and KEGG databases (Table S2).

**Table 2:** RNA-seq alignment details and mapping ratios

Samples	Total reads (M)	Mapped reads (M)	Uniq mapped reads (M)	Multiple map reads (M)	Reads map to ‘+’	Reads map to ‘-’
Non-infected rainbow trout 1	53.8382	45.6616 (84.81 %)	42.0247 (78.06 %)	3.6369 (6.76 %)	22.4170 (41.64 %)	22.6348 (42.04 %)
Non-infected rainbow trout 2	46.9109	40.1607 (85.61 %)	36.7008 (78.24 %)	3.4599 (7.38 %)	19.7468 (42.09 %)	19.9220 (42.47 %)
Non-infected rainbow trout 3	51.0477	43.3188 (84.86 %)	39.8427 (78.05 %)	3.4761 (6.81 %)	21.3261 (41.78 %)	21.4872 (42.09 %)
YR-infected rainbow trout 1	45.1922	38.7941 (85.84 %)	35.5522 (78.67 %)	3.2418 (7.17 %)	19.0209 (42.09 %)	19.2198 (42.53 %)
YR-infected rainbow trout 2	47.0682	40.4739 (85.99 %)	37.2354 (79.11 %)	3.2386 (6.88 %)	19.7989 (42.06 %)	20.0358 (42.57 %)
YR-infected rainbow trout 3	51.4973	43.9318 (85.31 %)	40.4383 (78.53 %)	3.4934 (6.78 %)	21.4897 (41.73 %)	21.7333 (42.20 %)
Non-infected group	50.5989	129.1411 (85.09 %)	118.5682 (78.12 %)	10.5729 (6.98 %)	63.4899 (41.84 %)	64.0440 (42.20 %)
YR-infected group	47.9192	123.1998 (85.71 %)	113.2259 (78.77 %)	9.9738 (6.94 %)	60.3095 (41.96 %)	60.9889 (42.43 %)
Total	295.5624	252.3409 (85.40 %)	231.7941 (78.44 %)	20.5467 (6.96 %)	123.7985 (41.90 %)	125.0329 (42.32 %)

### DEG identification and analysis

The Pearson’s correlation coefficient values were used to assess relative gene expression in the uninfected and YR-infected groups (Fig. S1). A total of 2498 DEGs were identified by comparing these groups, of which 2083 (83.39 %) were up-regulated and 415 (16.61 %) were down-regulated, in YR-infected fish compared to uninfected fish (Table S3). Volcano and MA plots were also used to represent these gene expression trends (Fig. S2).

Of these DEGs, 2431 were classified successfully using the NR, Swiss-Prot, GO, COG, KOG, Pfam, and KEGG databases (Table 3). With respect to new genes, many DEGs were annotated using the NR and eggNOG databases, but few were annotated in the COG database.

**Table 3:** Summary statistics regarding DEG functional annotation

Annotated databases	NR	Swiss-Prot	GO	COG	KOG	Pfam	KEGG	eggNOG	All
DEGs number	2421	1679	1766	584	1539	2116	1533	2295	2431
Ratio (%)	99.59	69.07	72.65	24.02	63.31	87.04	63.06	94.41	

**Fig. 2:**
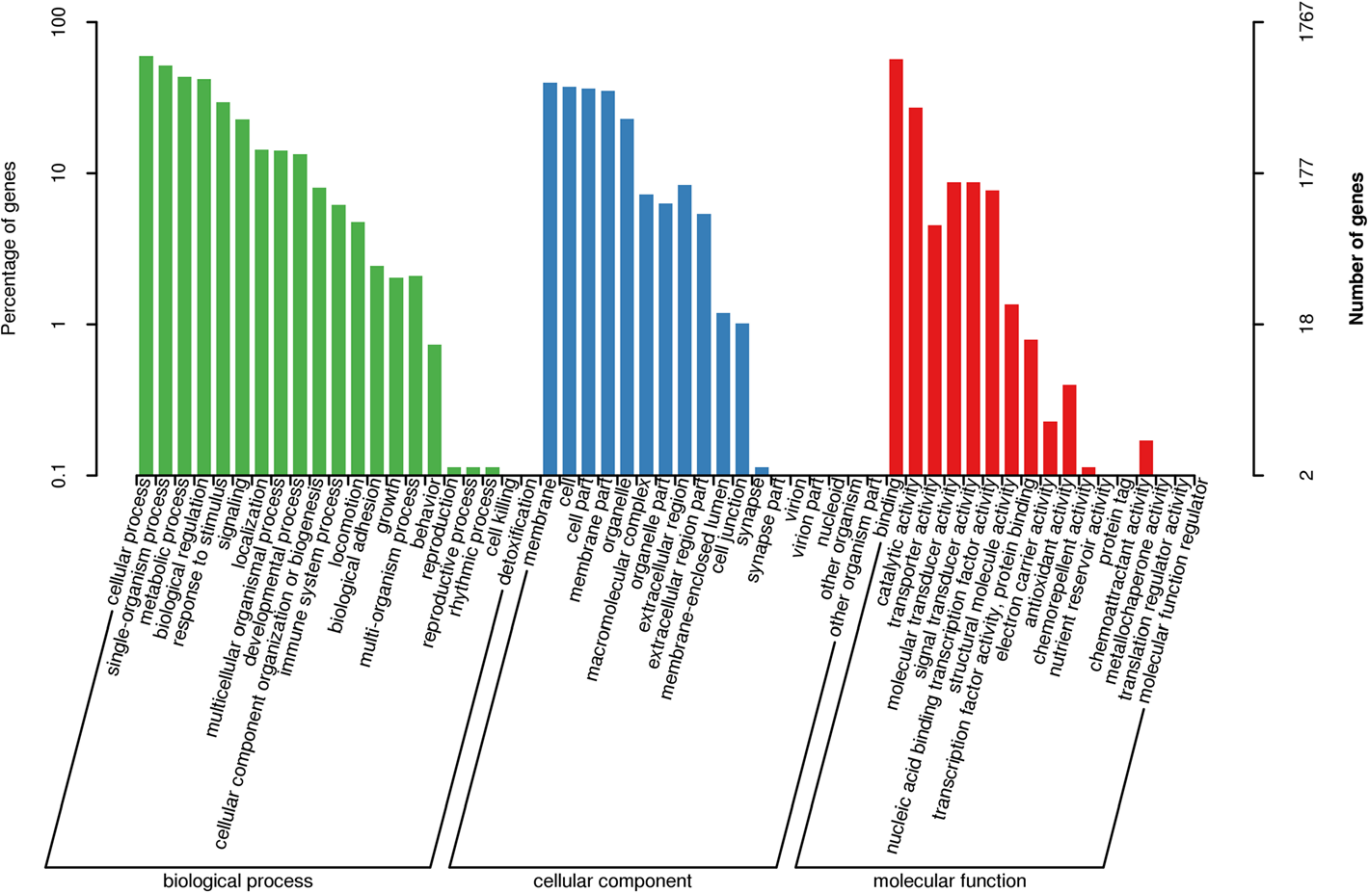
GO annotation of DEGs. DEGs were classified based on their enrichment in specific biological processes, cellular components, and molecular functions

To better understand the functional roles of detected DEGs, GO annotation was next performed by categorizing these DEGs into 23 biological processes (BPs), 19 cellular components (CCs), and 16 molecular functions (MFs). Cellular (42.07 %), single-organism (36.51 %), metabolic (30.75 %), and biological (29.64 %) processes were the most dominant categories of BPs, while membrane (27.94 %), cell (26.34 %), cell part (25.66 %), and membrane part (24.70 %) were the most enriched CCs and binding (40.07 %) and catalytic activity (19.26 %) were the most dominant MFs (Fig. 2).

**Fig. 3:**
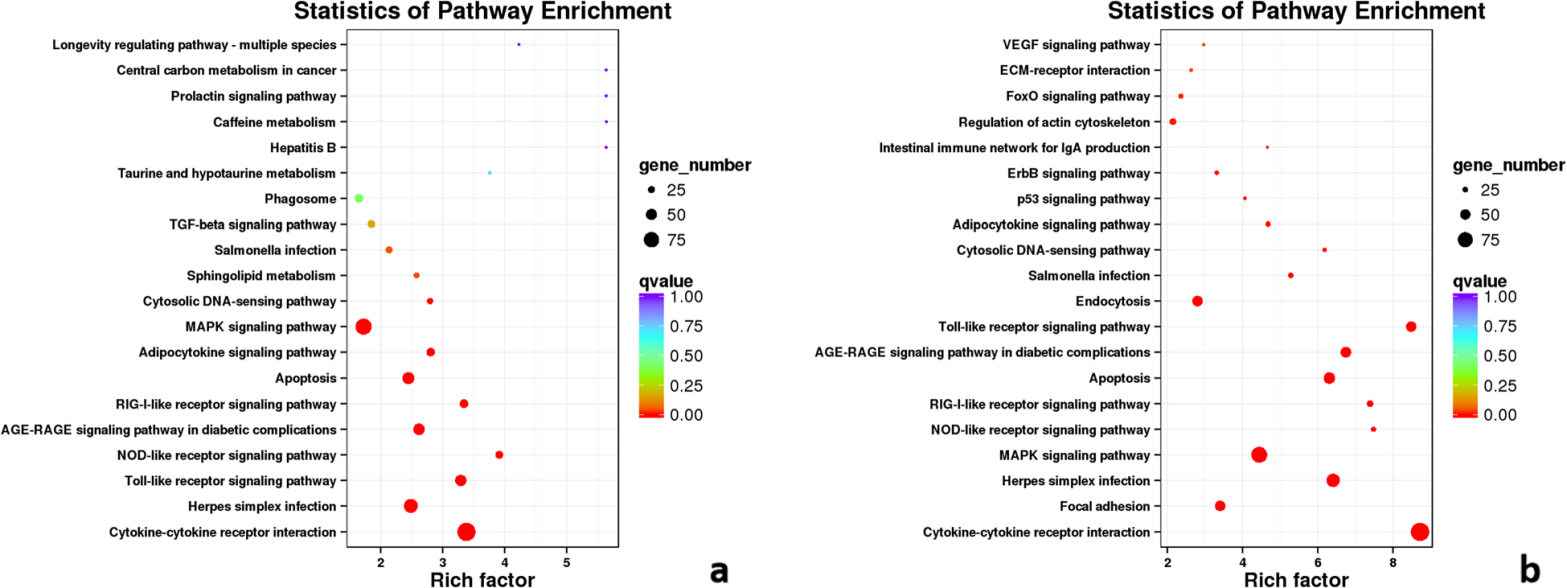
KEGG pathway enrichment results. Rich factor corresponds to the ratio of the total DEGs relative to total genes in the indicated pathways. **a** KEGG pathway enrichment results for all DEGs. **b** KEGG pathway enrichment results for those DEGs only involved in the top 9 pathways

In addition, KEGG pathway enrichment analyses were performed to assess the functional roles of these DEGs during *Y. ruckeri* infection in rainbow trout. Assembled DEGs were analyzed with the KEGG database, leading to their classification into 6 categories (Fig. S3). KEGG enrichment results, including the top 9 pathways enriched for > 50 genes (*P* < 0.05), are shown in Fig. 3. Four highly enriched pathways were detected through this KEGG analysis, including the NOD-like receptor signaling, cytokine-cytokine receptor interaction, Toll-like receptor signaling, and RIG-I-like receptor signaling pathways. The preferential enrichment of these pathways suggests that many of the genes differentially expressed between uninfected and YR-infected rainbow trout were related to the immune system.

### Identification of immune‐related DEGs

**Fig. 4:**
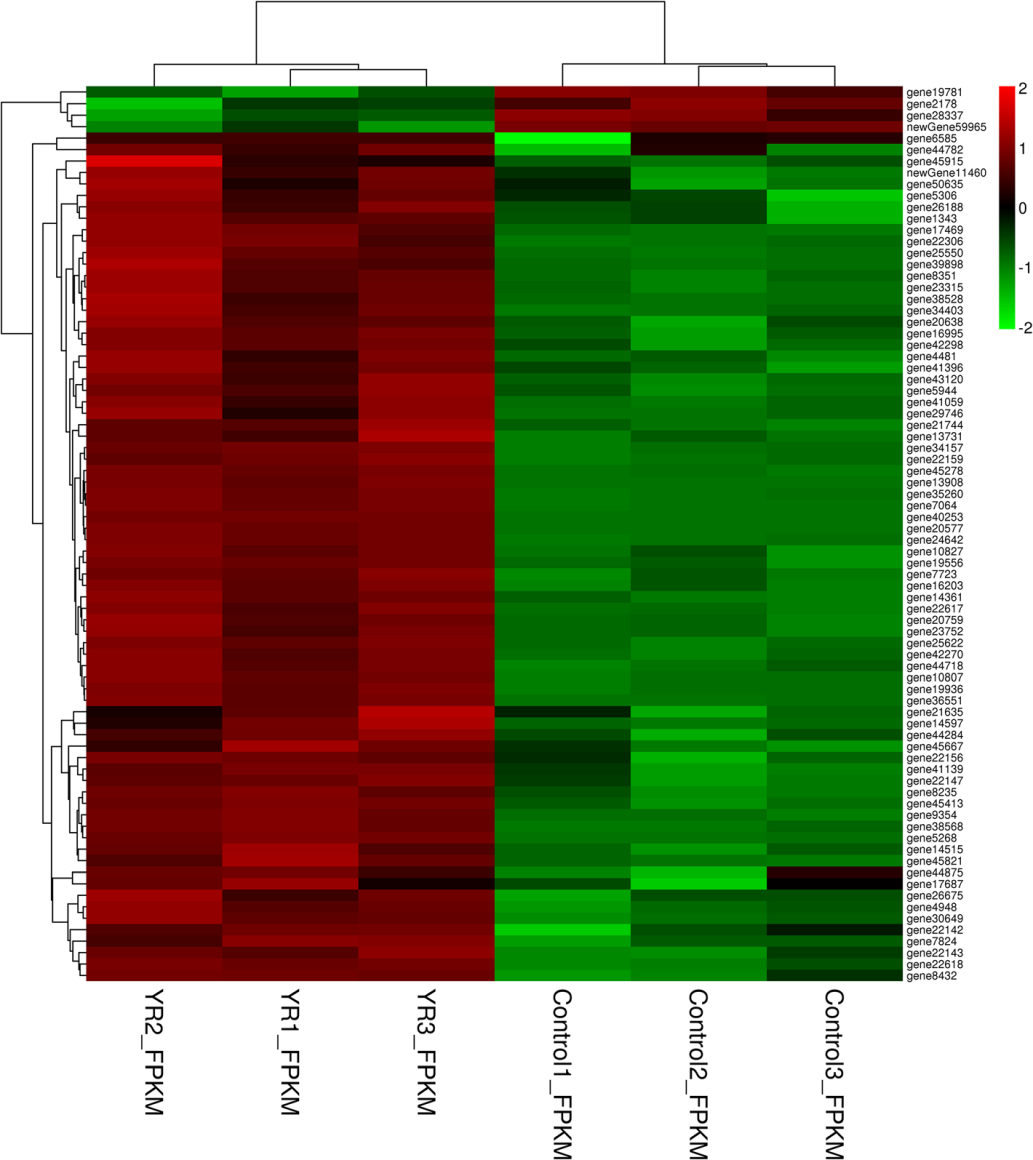
Immune-related DEGs in the non-infected and YR-infected rainbow trout

To better understand the intracellular signaling pathways during *Y. ruckeri* infection in rainbow trout, we therefore focused on 78 immune response-related DEGs identified in this study, including two new genes (Table S4). A heatmap was constructed based upon the fold-change expression values for these DEGs (Fig. 4), clearly demonstrating that almost all of these genes (74) were upregulated in spleen samples from YR-infected fish compared to spleen samples from uninfected fish, whereas only 4 genes were down-regulated after infection.

**Fig. 5:**
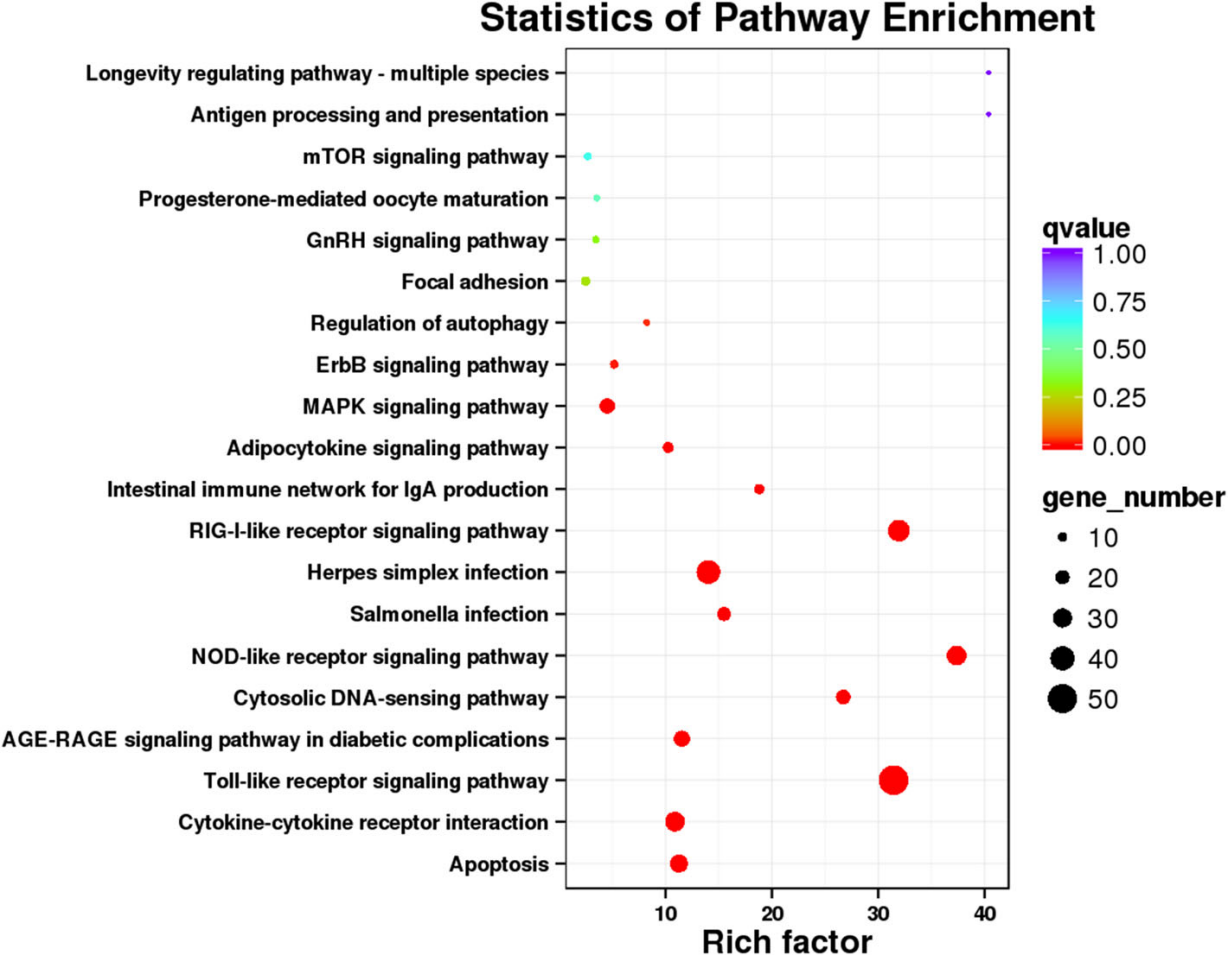
KEGG pathways enriched in genes differentially expressed between uninfected and YR-infected rainbow trout

Further analysis of these immune-related DEGs revealed them to be primarily associated with 20 immunological KEGG pathways, including the MAPK signaling, Cytokine-cytokine receptor interaction, Toll-like receptor signaling, RIG-I-like receptor signaling, NOD-like receptor signaling, FoxO signaling, mTOR signaling, apoptosis, TGF-beta signaling, regulation of autophagy, ErbB signaling, cell adhesion molecule (CAM), intestinal immune network for IgA production, cytosolic DNA-sensing, phosphatidylinositol signaling system, and p53 signaling pathways (Table 4). The top 3 pathways enriched in these genes included the NOD-like receptor signaling (31 genes), RIG-I-like signaling (35 genes), and Toll-like receptor signaling (51 genes) pathways (Fig. 5).

**Table 4:** Immune-related DEGs

Gene ID	Type	Log_2_Fold	Putative homolog protein	KEGG pathway
Gene 10,807	up	12.0338	Interleukin-1 beta	ko04620: Toll-like receptor signaling pathway
Gene 24,642	up	10.6682	Interleukin-8	ko04060: Cytokine-cytokine receptor interaction
Gene 22,618	up	9.3812	Interleukin-8	ko04621: NOD-like receptor signaling pathway
Gene 4948	up	8.4892	Tumor necrosis factor	ko04150: mTOR signaling pathway
Gene 28,337	down	-2.5818	Mitogen-activated protein kinase 11	ko04010: MAPK signaling pathway
Gene 25,622	up	10.1562	Interleukin-6	ko04060: Cytokine-cytokine receptor interaction
Gene 34,157	up	9.0084	Interleukin-6	ko04060: Cytokine-cytokine receptor interaction
Gene 34,403	up	6.5996	Tumor necrosis factor	ko04060: Cytokine-cytokine receptor interaction
Gene 25,550	up	6.1699	Tumor necrosis factor	ko04060: Cytokine-cytokine receptor interaction
Gene 22,142	up	3.1333	Interferon	ko04060: Cytokine-cytokine receptor interaction
Gene 2178	down	-1.1786	NOD1	ko04621: NOD-like receptor signaling pathway
Gene 20,638	up	4.6720	Small cytokines (intecrine/chemokine)	ko04060: Cytokine-cytokine receptor interaction
newGene59965	down	-1.4640	Toll-like receptor 8	ko04620: Toll-like receptor signaling pathway
Gene 26,188	up	3.7094	Mab-21 protein	ko04623: Cytosolic DNA-sensing pathway
Gene 44,284	up	3.1644	Immunoglobulin V-set domain	ko04514: Cell adhesion molecules (CAMs)
Gene 23,752	up	2.9294	Phosphoinositide 3-kinase regulatory subunit	ko04012: ErbB signaling pathway
Gene 5944	up	2.4306	Interferon alpha/beta receptor	ko04060: Cytokine-cytokine receptor interaction

### Validation of selected DEGs by qRT-PCR

**Fig. 6:**
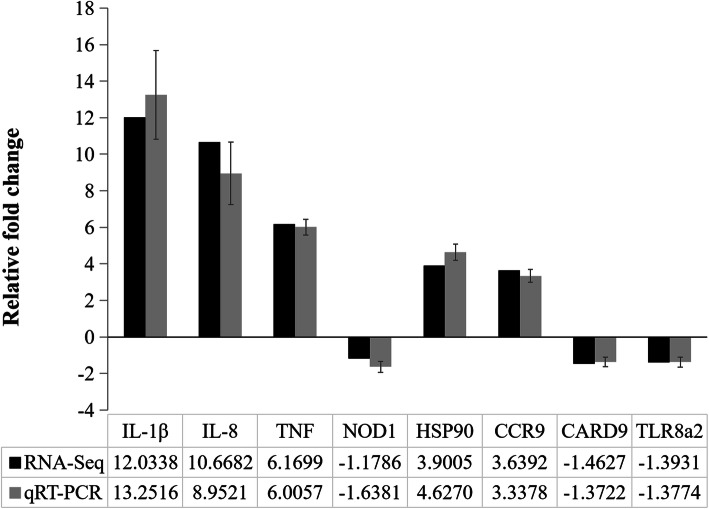
Comparison of DEG expression in qPCR and RNA-seq analyses. Relative gene expression levels were normalized to *EF-1α*

As expected, all the eight immune-related DEGs exhibited similar expression trends when measured via both qPCR and RNA-Seq analysis, confirming the reliability of our analytical techniques (Fig. 6).

## Discussion

ERM is a serious disease that impacts global salmonid populations [14]. While some studies have begun to characterize rainbow trout immune responses to *Y. ruckeri* infection [8, 15], no systematic transcriptomic analyses of these responses have been conducted to date. The spleen plays central roles in orchestrating innate and adaptive immune responses in fish. Herein, we sequenced the spleen transcriptomes of rainbow trout infected with YR in comparison with those of control uninfected rainbow trout and we identified 2498 DEGs between these populations, of which 2083 were up-regulated whereas 415 were down-regulated in infected rainbow trout. Immune response-related DEGs were then assessed in additional detail in an effort to explore the basis of immune responses against *Y. ruckeri* infection in rainbow trout.

Cytokines are secreted by a range of cell types, and they act as immune response regulators that can be classified as interleukins (ILs), interferons (IFNs), tumor necrosis factors (TNFs), and chemokines [16]. Of the 78 immune-associated DEGs in the present study, 31 were classified into the cytokine-cytokine receptor interaction pathway, including chemokine (C-X-C motif) ligand (*CXCL11*), C-C motif chemokine receptor 9 (*CCR9*), caspase recruitment domain-containing protein (*CARD9*), *IL-12*, *IL-1β*, *IFN* and *TNF*. Chemokines control the migration of particular immune cell subsets and coordinate both adaptive and innate immune responses to stressors [17]. The transcription of *CXCd* in rainbow trout has previously been shown to be induced in response to *Y. ruckeri* infection [18]. Herein, we observed the upregulation of both *CXCL11* and *CCR9* in the spleens of rainbow trout infected with this bacterium, consistent with the pathogen-induced chemokine regulation. *CARD9*, which is normally activated by CLRs [19], was 1.39-fold downregulated in response to *Y. ruckeri*. Zuo et al. [8] investigated the immune gene expression in rainbow trout to *Y. ruckeri* infection by qRT-PCR and indicated that the genes encoding inflammatory cytokines (*IL-1β*, *IL-2 A*, *IL-6 A*, *IL-8*, *IL-10 A*, *IL-12*, *IL-17 A/F2A*, *IL-17C1*, *IL-17C2*, *IL-22*, *TNFα*) were generally upregulated in spleen, gills and liver. Our findings also showed the same results on the cytokines expression during *Y. ruckeri* infection, suggesting involvement of these immune-related genes in response of rainbow trout to bacterial infection (Table 4).

Apoptosis is an important determinant of cellular survival in both physiological and pathological contexts, and can be triggered by factors such as hypoxia, chemical exposure, temperature stress, or immune responses to particular stimuli. Upon bacterial infection, a host’s cells may undergo apoptotic death to mitigate the spread of the pathogen within host tissues [20]. Herein, we observed the upregulation of caspase 8 (*CASP8*), receptor-interacting serine/threonine-protein kinase 1-like (*RIPK1*) and NF-kappa-B inhibitor alpha-like (*IκBα*) following YR infection in rainbow trout. Caspases are proteases that serve as essential regulators of apoptotic cell death, with *CASP8* having showed to be an upstream regulator of apoptotic cascades in fish [21]. Marked *CASP8* upregulation has also previously been detected in head-kidney and spleen leukocytes of *Totoaba macdonaldi* at 24 h post-infection with *Vibrio parahaemolyticus* and *Aeromonas veronii* [22]. *RIPK1* was identified as a central driver of inflammation in atherosclerosis by its ability to activate the *NF-κB* pathway and promote inflammatory cytokine release in mice (*Mus musculus*) [23]. *NF-κB* can control innate and adaptive immune-related gene expression, inducing apoptosis in response to numerous stimuli [24]. At the same time, *NF-κB* activation induces *IκBα* expression in rainbow trout, in turn resulting in the feedback inhibition of *NF-κB* [25]. Upregulation of *IkBα*, *IAPs* and *RIPK1* detected in this study can suggest the compensatory activation of some inhibitors of apoptotic cell death, underscoring the complexities of cellular responses to *Y. ruckeri* in rainbow trout. Additional work must be done in order to understand in depth how the apoptotic processes.

Pattern recognition receptors (PRRs) serve as innate sensors that can rapidly detect and respond to conserved damage- and pathogen-associated molecular patterns (DAMPs and PAMPs, respectively), resulting in the induction of immune-related gene expression and anti-pathogen responses. PRRs detected in aquatic species to date include TLRs, NLRs, RLRs, and CLRs [26]. In the present study, we identified several DEGs belonging to TLR, NLR, and RLR gene families in the spleens of rainbow trout at 24 h post-*Y. ruckeri* infection, including nucleotide-binding oligomerization domain-containing protein 1-like (*NOD1*), toll-like receptor 8α2 (*TLR8α2*), etc. *NOD1* modulates the innate immune response of fish to bacterial peptidoglycan. Loss- and gain-of-function experiments have suggested that *NOD1* can control rainbow trout pro-inflammatory cytokines in rainbow trout [27]. Palti et al. first reported the presence of the *TLR8α2* gene in rainbow trout, which they found to be somewhat downregulated in response to treatment with the human agonist of TLR7/8 known as R848 [28]. Here we found that both *NOD1* and *TLR8α2* were downregulated in rainbow trout spleen during the early stages of *Y. ruckeri* infection. KEGG pathway analysis indicated that many DEGs were involved in TLR signaling pathway, NLR signaling pathway and RLR signaling pathway, such as heat shock protein 90 (*HSP90*), tumor necrosis factor alpha-induced protein 3-like (*TNFAIP3*), transcription factor AP-1, *IL-1*, *IL-12*, *NF-κB*, *RIPK1*, *CASP8* and so on. HSPs are important regulators of fish immune responses [29, 30], and *HSP90* upregulation detected in the present research may be linked to the rainbow trout innate immune defenses to *Y. ruckeri* infection. Of interest, a pathogen-specific expression pattern of *HSP90* was observed in channel catfish (*Ictalurus punctatus*) and it showed different expression patterns following *Flavobacterium columnare* and *Edwardsiella ictaluri* infection [31]. *TNFAIP3* (A20) acts as a negative feedback regulator of RIG-I pathway for the establishment of an antiviral state in teleost. *TNFAIP3* interrupted RIG-I signaling at the level of TBK1 kinase, a critical point of convergence for many different pathways that activates important transcription factors involved in the expression of many cytokines [32]. In the present study, *TNFAIP3* was found to be upregulated after *Y. ruckeri* infection, demonstrating that this gene was involved in immune response of rainbow trout during bacterial infection. Overall, these findings suggest that the PRRs were differentially expressed in rainbow trout and may be important mediators of the initial induction of immunological responses to bacterial infection.

The MAPK signaling pathway is responsive to diverse extracellular stimuli and can modulate transcription factor expression and activation, controlling a range of biological processes including proliferation, apoptosis, and gene transcription. Recent evidence indicates that fish MAPKs can be induced by a range of stimuli. For example, flagellin treatment is associated with *MAPK11* upregulation in the head kidney of rock bream (*Oplegnathus fasciatus*) [33]. In contrast, in the present study we observed a 2.58-fold decrease of *MAPK11* expression in the spleen of rainbow trout following *Y. ruckeri* infection, although additional validation of these results is warranted. *MAPK8* (*Jnk1*) has been identified in many fish species and its expression pattern varies by different stimuli [34, 35]. Infection with *Aeromonas hydrophila* and *Bacillus subtilis* could induce significant expressions of the jnk1 gene in *Labeo rohita* [36]. We also observed a significant *MAPK8* upregulation upon *Y. ruckeri* infection in rainbow trout, suggesting that these MAPKs might play a crucial role during the bacterial pathogenesis in rainbow trout. Moreover, a total of 22 DEGs involved in the MAPK signaling pathway seems to play key roles in the rainbow trout response to infection with this bacterium.

## Conclusions

In summary, we conducted a transcriptomic analysis of spleen samples from rainbow trout infected with *Y. ruckeri* in an effort to better understand the immunological basis for responses to this pathogen, leading to the identification of several key immune-related DEGs. Overall, our results will provide a preliminary insight on the immune responses of rainbow trout following *Y. ruckeri* infection and the base for future studies of host-pathogen interactions in rainbow trout.

## Methods

### Experimental fish and bacteria

Healthy rainbow trout (~ 10 g) were obtained from Benxi Agrimarine Industries Inc. and maintained in a 540 L fiberglass circulating water tank at a constant temperature of 14 ± 0.2℃ with a 12 h light/dark cycle and an 8.0 mg/L oxygen saturation. Fish were maintained under these conditions for 2 weeks and were fed commercial rainbow trout feed.

*Y. ruckeri* strain BH1206 was isolated from infected rainbow trout, confirmed to be pathogenic, and used for challenge experiments as previously published [37]. Bacteria were grown for 24 h in TSB medium (BD Difco, USA) and collected by spinning for 5 min at 6,000 xg prior to resuspension in sterile PBS (pH 7.2) at 6 × 10^7^ CFU·mL^− 1^.

### Bacterial challenge and sampling

 Prior to challenge test, healthy rainbow trout were kept under laboratory conditions in flow-through tanks at approximately 14 °C with continuous aeration and fed twice a day at 1.2 % of body weight with commercial fish feed. A subset of experimental fish was microscopically and bacteriologically examined to verify freedom of *Y. ruckeri* infection. Tricaine methanesulfonate (MS222) was used to anesthetize fish prior to the challenge or tissue sample collection. For the challenge test, the experimental fish were kept in two tanks with 6 fish in each tank under the same conditions for fish acclimation. Experimental infection was induced by intraperitoneally (i.p.) injecting fish with 100 µL of BH1206 bacteria at 6 × 10^5^ CFU per gram of fish body weight. An equivalent volume of PBS was injected into uninfected control fish. At 24 h post-infection, three fish per group were sacrificed by an overdose of anesthetic, and spleens were collected, washed to remove blood and fat, snap-frozen with liquid nitrogen, finally stored in liquid nitrogen tank. To confirm the presence of *Y. ruckeri* in experimental fish, the kidney was sampled to perform bacteriological examination.

### RNA isolation

Splenic RNA was isolated using Trizol (Invitrogen, USA), after which RNA integrity and purity were evaluated via 1 % agarose gel electrophoresis and using an Agilent 2100 Bioanalyzer (Agilent Technologies, CA, USA), while a Qubit RNA Assay Kit and a Qubit 2.0 Fluorometer (Life Technologies, CA, USA) were utilized to measure RNA concentration. After RNA preparation, all downstream library preparation and sequencing were performed by Biomarker technologies CO., LTD (Beijing, China).

### Library construction and sequencing

A total of 3 µg RNA per spleen sample was utilized for library construction using a NEBNext Ultra RNA Library Prep kit for Illumina (NEB, USA), with samples being affixed with appropriate barcodes. Following DNase I treatment, the remaining mRNA was purified and sheared into 200–250 bp fragments as discussed previously [38]. Library quality was assessed with an Agilent Bioanalyzer 2100 instrument, and a cBot Cluster Generation System with TruSeq PE Cluster Kit v4-cBot-HS (Illumina) was used to cluster barcoded samples. An Illumina Hiseq 2500 platform was then used for the paired-end sequencing of these prepared library samples.

### Data processing

Raw data were initially cleaned by removing reads that contained adapter sequences, poly-N sequences, and low-quality reads with the FastQC program (http://www.bioinforatics.babraham.ac.uk/projects/fastqc/), after which clean data Q30, GC-content, and sequence duplication levels were calculated. The Trinity software [39] was then used to assemble reads into EST clusters, followed by de novo assembly and alignment to the rainbow trout reference genome (http://www.genoscope.cns.fr/trout/data/) with TopHat (v.2.0.5). Functional annotation was performed by comparing unigenes to the following databases: Nr (NCBI non-redundant protein sequences); Nt (NCBI non-redundant nucleotide sequences);Pfam (Protein family); KOG/COG (Clusters of Orthologous Groups of proteins) [40]; Swiss-Prot (A manually annotated and reviewed protein sequence database); KO (KEGG Ortholog database) [41]; GO (Gene Ontology) [42].

### DEGs identification

The RSEM software was used to assess unigene expression based upon reads per kilobase of exon per million mapped reads (RPKM) [43]. The DESeq R package (1.10.1) was used to identify DEGs between infected and non-infected fish using a negative binomial distribution-based model, with *P* values being adjusted as indicated by the Benjamini and Hochberg approach to reduce the false discovery rate. DEGs were considered as those genes with an adjusted *P*-value < 0.05, and were represented with volcano and MA plots. The Kyoto Encyclopedia of Genes and Genomes (KEGG) database was used for functional enrichment analysis of DEGs, with pathways that had a Q-value ≤ 0.05 after correcting for multiple testing being considered significantly enriched [44–46]. Furthermore, the immune-related DEGs were selected by mapping the ‘5.1 Immune system’ in KEGG maps (https://www.genome.jp/kegg/pathway.html).

### Validation of immune‐related DEGs by qRT-PCR

To confirm the results of RNA-sequencing, eight immune-related DEGs (*IL-1β, IL-8, TNF, NOD1, CARD9, TLR8α2, CCR9*, and *HSP90*) were randomly selected for qRT-PCR-based validation using the same RNA samples prepared for RNA-seq using primers designed with the Premier primer 5 software (Table 5). *EF-1α* was used as a normalization control for these analyses. SYBR Green dye (Takara, China) and an ABI PRISM 7500 Fast Real-time PCR instrument were used for qRT-PCR based on provided protocols. All reactions were conducted in triplicate with the following thermocycler settings: 60 s at 95℃; 40 cycles of 15 s at 95℃, 45 s at 60℃. Melt curve analyses were conducted to confirm the specificity of amplification products. Relative gene expression was assessed via the 2^−△△CT^ approach [47].

**Table 5:** qRT-PCR Primers Lists

Gene_ID	Gene name	Forward primer sequence (5’-3’)	Reverse primer sequence (5’-3’)
Gene10807	*IL-1β*	CAACTAAGATGGCCGCAAA	TCGGTACATACTCTAAACCTC
Gene24642	*IL-8*	ATTTATAAGCTTGATAGGCTG	GTTGTATATAAGAAACCGACT
Gene25550	*TNF*	CAGGAGCATCACTACCTTC	TTACTAGAACTTTCTGCGGAT
Gene2178	*NOD1*	ATACAACTGCTACCCCGACCA	AGGCACATTCACCAGGTCCA
Gene14361	*HSP90*	GATCCTTCACCGTCAAAGTCG	TCACTTCCTTGTCACGCTCC
Gene45413	*CCR9*	ATCTTGAATTTAAGCGCCTGT	ACATCATCCTCACCAACCGTA
Gene19781	*CARD9*	TGACAACACTGACACGGAT	ATGCACATGAAGAGATACAAGC
newGene59965	*TLR8a2*	CTCTGCCATTTTGATTGGGA	CCCCTAAGAAATCCACGAGA
Housekeeping gene	*EF-1α*	GATCCAGAAGGAGGTCACCA	TTACGTTCGACCTTCCATCC

## Supplementary Information

**Additional file 1.**

**Additional file 2.**

**Additional file 3.**

**Additional file 4: Table S1.** Characteristics of RNA-seq data.

**Additional file 5: Table S2.** Summary of information regarding the annotation of new genes.

**Additional file 6: Table S3.** Information of all the DEGs detected in this study.

**Additional file 7: Table S4.** The list of immune response-related DEGs identified in this study.

## Data Availability

The dataset(s) supporting the conclusions of this article is(are) included within the article (and its additional file(s). The raw data were available in the NCBI Sequence Read Archive (SRA).

## Abbreviations

YR: *Yersinia ruckeri*
DEGs: Differentially expressed genes
GO: Gene ontology
KEGG: Kyoto encyclopedia of genes and genomes
ILs: Interleukins
IFNs: Interferons
TNFs: Tumor necrosis factors
CCR9: C-C motif chemokine receptor 9
CARD9: Caspase recruitment domain-containing protein
CASP8: Caspase 8
RIPK1: Receptor-interacting serine/threonine-protein kinase 1-like
IAP: Inhibitor of apoptosis protein-like protein
*IκBα*: NF-kappa-B inhibitor alpha-like
PPRs: Pattern recognition receptors
HSP90: Heat shock protein 90
NOD1: Nucleotide-binding oligomerization domain-containing protein 1-like
TLRs: Toll-like receptors
TNFAIP3: Tumor necrosis factor alpha-induced protein 3-like
