# Supplementary figures and images for: Transcriptome profiling of immune response to Yersinia ruckeri in spleen of rainbow trout (Oncorhynchus mykiss)
Source: BMC Genomics. 2021 Apr 22;22:292. doi: 10.1186/s12864-021-07611-4 (PMC8061174; doi:10.1186/s12864-021-07611-4)

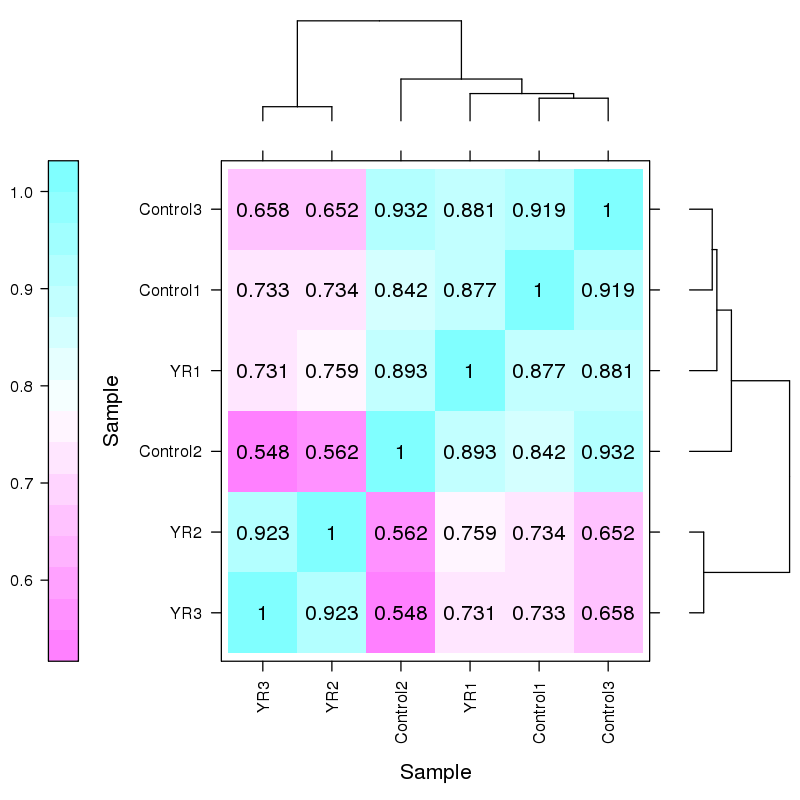

Supplement: Supplementary file 1 — Additional file 1. [file 12864_2021_7611_MOESM1_ESM.png]

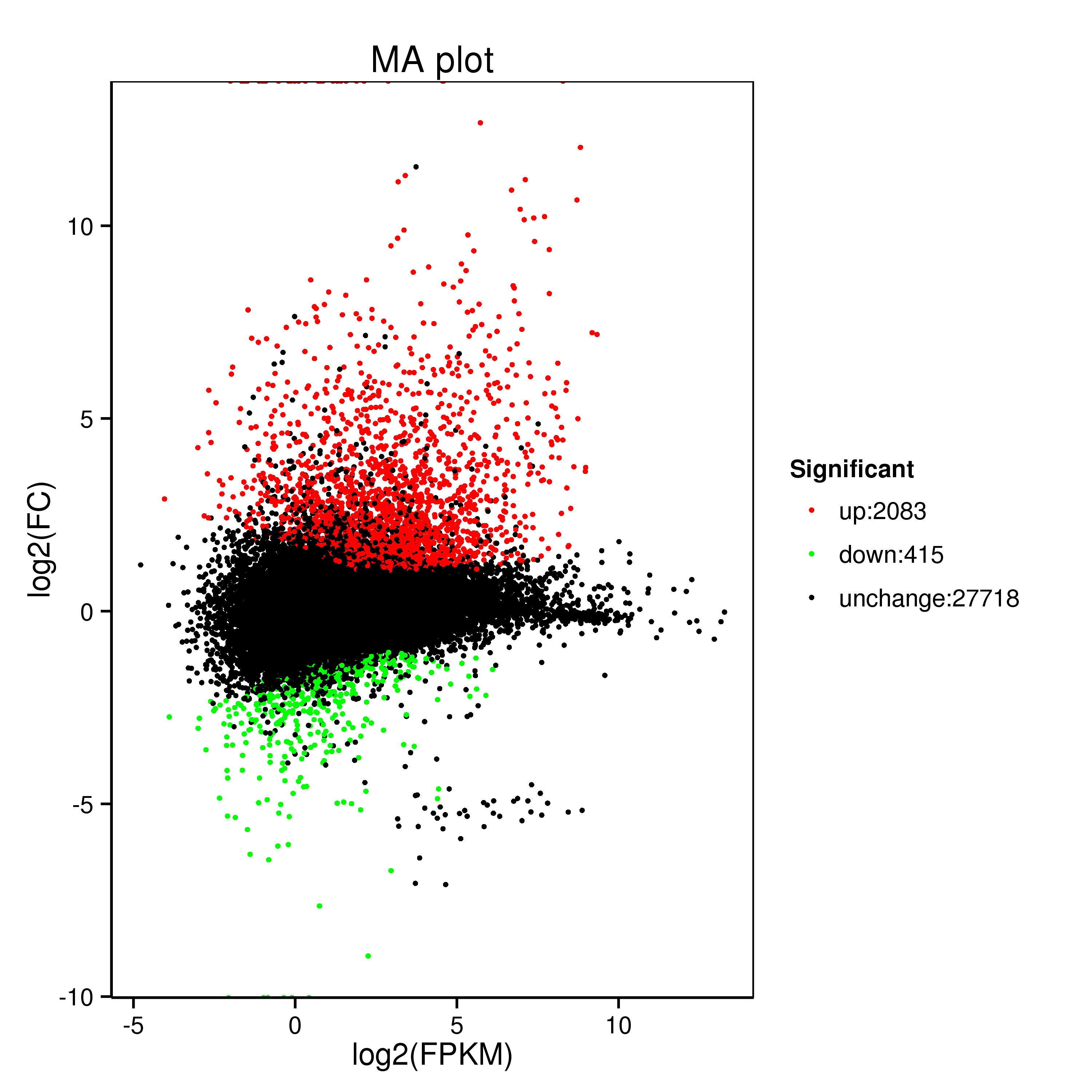

Supplement: Supplementary file 2 — Additional file 2. [file 12864_2021_7611_MOESM2_ESM.zip › Fig. S2A Volcano plot of DEGs between YR-infected group and control group.png]

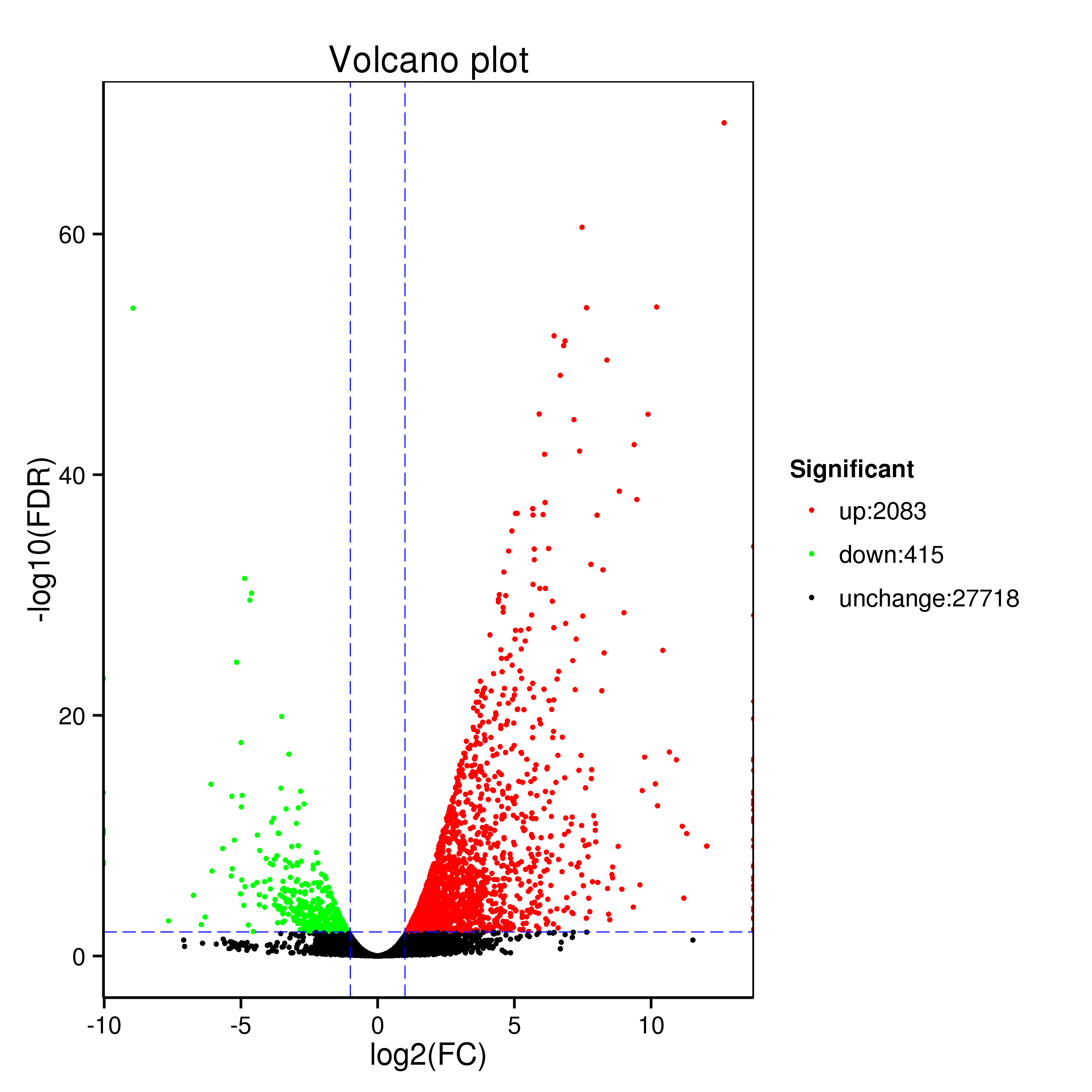

Supplement: Supplementary file 2 — Additional file 2. [file 12864_2021_7611_MOESM2_ESM.zip › Fig. S2B MA plot of DEGs between YR-infected group and control group.png]
